# Extracellular ATP increases agonist potency and reduces latency at class B G protein-coupled receptors

**DOI:** 10.1016/j.molpha.2025.100040

**Published:** 2025-04-22

**Authors:** Shuying Zhu, Alice Yuan, Tristan Duffy, Brandon H. Kim, Takeaki Ozawa, S. Jeffrey Dixon, Peter Chidiac

**Affiliations:** 1 Department of Physiology and Pharmacology, Schulich School of Medicine & Dentistry, The University of Western Ontario, London, Canada; 2 Department of Anesthesiology, Sichuan Clinical Research Center for Cancer, Sichuan Cancer Hospital and Institute, Sichuan Cancer Center, Affiliated Cancer Hospital of University of Electronic Science and Technology of China, Chengdu, China; 3 Bone and Joint Institute, The University of Western Ontario, London, Canada; 4 Department of Chemistry, School of Science, University of Tokyo, Tokyo, Japan

**Keywords:** Adenosine 5'-triphosphate, Adenylyl cyclase, *β*-Arrestin, Class B GPCR, G protein-coupled receptor, Lag time, Potentiation

## Abstract

Class B G protein-coupled receptors (GPCRs) are peptide hormone receptors, many of which, such as parathyroid hormone receptor 1, calcitonin receptor (CTR), and corticotropin-releasing factor receptor (CRF1R), are established or emerging therapeutic targets. Previously, we showed that extracellular ATP and related molecules act as positive modulators of parathyroid hormone receptor 1 signaling through an undefined mechanism. Here, we investigated whether ATP enhances signaling by other members of the class B family of GPCRs. Cyclic AMP (cAMP) accumulation was monitored in cells expressing a bioluminescent sensor. Extracellular ATP, which did not induce cAMP accumulation on its own, potentiated agonist-induced cAMP accumulation mediated by CTR, CRF1R, calcitonin receptor-like receptor, pituitary adenylyl cyclase-activating polypeptide receptor 1, and vasoactive intestinal peptide receptors 1 and 2. ATP induced a comparable effect on agonist-stimulated recruitment of *β*-arrestin to pituitary adenylyl cyclase-activating polypeptide receptor 1. Depending on the receptor and agonist, ATP increased agonist potency by up to 50-fold. The enhancing effect of ATP was mimicked by cytidine 5'-monophosphate, ruling out involvement of purinergic receptors, ATPase activity, or ectokinase activity. For certain receptors (CTR, calcitonin receptor-like receptor + receptor activity-modifying protein 1, and CRF1R), there were temporal lags of up to 30 minutes following agonist application before maximal rates of cAMP accumulation were reached. Lag duration decreased with increasing agonist concentration, suggesting an inverse relationship with receptor occupancy. ATP virtually abolished this temporal lag, even at relatively low agonist concentrations. Thus, ATP both increases the potency of orthosteric agonists at class B GPCRs and reduces latency for adenylyl cyclase activation.

**Significance Statement:**

In addition to acting as a positive modulator of PTH1R signaling, extracellular ATP increases the potency of orthosteric agonists at other class B GPCRs and reduces the latency for adenylyl cyclase activation. Further insight into the precise mechanism of ATP-mediated potentiation of class B GPCR signaling may identify new targets for the development of therapeutic agents aimed at the treatment of endocrine disorders.

## Graphical abstract

**Figure:**
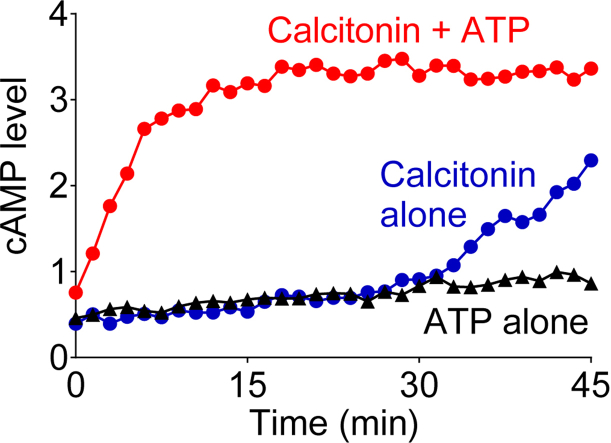

## 1 Introduction

Class B or secretin-like receptors are a family of 15 G protein-coupled receptors (GPCRs) that are activated by a conserved family of endogenous peptide agonists. These receptors are implicated in developmental and homeostatic processes in the endocrine, skeletal, and central nervous systems (Karageorgos et al, 2018). Pharmacotherapeutics targeting class B GPCRs have proliferated in recent years and their place in the health care market is expected to expand (Bodenner et al, 2007; Vannabouathong et al, 2022). Class B receptors predominantly couple to G_s_ to activate adenylyl cyclase, but some can also interact with other G protein heterotrimers and *β*-arrestins to activate additional pathways such as phospholipase C*β* and MAPK/ERK (Gurevich and Gurevich, 2019).

ATP is released to the extracellular milieu in response to mechanical stimuli or coreleased from presynaptic vesicles with other signaling factors (Mikolajewicz et al, 2018; Lu et al, 2019). Extracellular nucleotides are known for their ability to act as purinoceptor agonists (Burnstock, 2007). We previously reported that extracellular ATP increases agonist potency at parathyroid hormone 1 receptor (PTH1R), a class B GPCR. Specifically, ATP was found to strongly enhance agonist-induced cAMP elevation and *β*-arrestin recruitment (Kim et al, 2018). Extracellular ATP had no effect on PTH1R signaling in the absence of added agonist, and its potentiating effects in the presence of agonist could not be ascribed to P2 purinergic receptor activation, ATPase activity, or ectokinase activity.

Because class B GPCRs bear sequence and structural homology to one another (Karageorgos et al, 2018), we reasoned that that the enhancing effect of ATP might not be limited to PTH1R. Thus, we investigated whether ATP might potentiate other class B GPCRs.

In the present study, we demonstrated that the presence of extracellular ATP potentiates signaling of multiple class B GPCRs, including the calcitonin receptor; the calcitonin gene–related peptide (CGRP) and adrenomedullin (AM) receptors (ie, calcitonin receptor-like receptor [CRLR] + receptor activity-modifying protein 1 [RAMP1; CGRP R], RAMP2 [AM_1_ R], or RAMP3 [AM_2_ R] [Born et al, 2002]); the corticotropin-releasing factor 1 receptor (CRF1R); and the receptors for pituitary adenylyl cyclase–activating polypeptide and vasoactive intestinal peptide (PAC1R, VPAC1R, and VPAC2R). ATP, although having little or no effect on its own, enhanced cAMP signaling via each of the aforementioned receptors. Some of the class B GPCRs tested in this study, namely, the calcitonin, CGRP, amylin, and CRF1 receptors, exhibited hysteresis in signaling kinetics wherein lag times were observed between agonist exposure and attainment of maximal rates of cAMP accumulation. In contrast, such delays were minimal or absent with PAC1R, VPAC1R, or VPAC2R. Notably, little or no hysteretic effect could be detected with any of the receptors tested when ATP was coadministered together with the agonist. Taken together, these findings suggest that ATP enhances agonist-induced activation of class B GPCRs both by increasing the rate of cAMP production and by reducing signaling latency.

## 2 Materials and methods

### 2.1 Materials and solutions

*α*-Minimum essential medium (*α*-MEM), heat-inactivated fetal bovine serum (FBS), antibiotic-antimycotic solution (10,000 U/mL penicillin, 10,000 *μ*g/mL streptomycin, and 25 *μ*g/mL amphotericin B), trypsin solution, Dulbecco’s phosphate-buffered saline, Dulbecco’s modified Eagle’s medium (high glucose) (DMEM), 2-[4-(2-hydroxyethyl)piperazin-1-yl]ethanesulfonic acid (HEPES), and minimum essential medium (MEM; without bicarbonate, and with or without phenol red) were obtained from Thermo Fisher Scientific. X-tremeGENE 9 reagent was from Roche Diagnostics. Bovine albumin (BSA)/Fraction V was obtained from MP Biomedicals. Adenosine 5'-triphosphate (ATP) disodium salt hydrate, cytidine 5'-monophosphate (CMP) disodium salt, and 3-isobutyl-1 methylxanthine (IBMX) were obtained from Sigma–Aldrich. Salmon calcitonin, pituitary adenylate cyclase–activating peptide (PACAP-38), and vasoactive intestinal peptide (VIP) were purchased from Bachem. Human *α*-calcitonin gene–related peptide was from Phoenix Pharmaceuticals. Recombinant human/rat CRF was obtained from Tocris Bioscience. d-luciferin sodium salt was obtained from Gold Biotechnology. Nucleotides were dissolved and diluted in divalent cation-free buffer: 140 mM NaCl, 5 mM KCl, 20 mM HEPES, and 10 mM glucose; pH = 7.30 ± 0.02, 290 ± 5 mOsmol/L (Veh_2_). Peptides were dissolved and diluted in Dulbecco's phosphate-buffered saline supplemented with 0.1% BSA (Veh_1_).

### 2.2 Plasmids

*CALCR* in pcDNA3.1(+) (encoding calcitonin receptor), *CARCRL* in pcDNA3.1(+) (encoding calcitonin receptor-like receptor, CRLR), *ADCYAP1R1* in pcDNA3.1(+) (encoding pituitary adenylate cyclase–activating polypeptide receptor type 1, PAC1R), *VIPR1* in pcDNA3.1(+) (encoding vasoactive intestinal polypeptide receptor 1, VPAC1R), *VIPR2* in pcDNA3.1(+) (encoding vasoactive intestinal polypeptide receptor 2, VPAC2R), and *CRHR1* in pcDNA3.1(+) (encoding CRF1R) were purchased from cDNA Resource Center. pGloSensor-22F cAMP plasmid was obtained from Promega. Plasmid *PtGRN415-ARRB1* in pcDNA3.1/Myc-His (B) (encoding N-terminal click beetle luciferase (1-415)-*β*-arrestin-1 chimeric protein) was as described previously (Misawa et al, 2010) and was used in conjunction with *ADCYAP1R1-linker20-PtGRC394* in pcDNA4 V5-His (B) (encoding human PAC1R-C-terminal click beetle luciferase [394-542]). To generate the latter construct, full-length *ADCYAP1R1* was amplified by PCR using forward primer (with BamHI) and reverse primer (with XhoI). The PCR product was digested with the restriction enzymes and inserted into the plasmid of pc4_*SSTR2-ELucC* (described in the supporting information [Misawa et al, 2010]) using the same enzymes. RAMP1 in pcDNA3, RAMP2 in pcDNA3, and RAMP3 in pcDNA3 (encoding human receptor activity-modifying protein; RAMP1, 2, and 3, respectively) were generously provided by Dr Patrick Sexton (Monash Institute of Pharmaceutical Sciences, Australia).

### 2.3 Cells and culture

COS-7 African monkey kidney cells, the UMR-106 rat osteoblast-like cell line, MCF-7 breast cancer cells, and SaOS-2 human osteosarcoma cells were subcultured 2 to 3 times weekly and maintained at 37 °C and 5% CO_2_ in *α*-MEM supplemented with 10% FBS and antibiotic–antimycotic solution (final concentrations: penicillin 100 units/mL, streptomycin 100 μg/mL, and amphotericin B 0.25 *μ*g/mL). HEK293H cells were subcultured 2 to 3 times weekly and maintained at 37 °C and 5% CO_2_ in DMEM supplemented with 10% FBS and antibiotic–antimycotic solution. COS-7 cells do not endogenously express RAMP proteins (Hay and Pioszak, 2016). MCF-7 cells endogenously express the calcitonin receptor and downstream signaling components including adenylyl cyclase (Findlay et al, 1981). SaOS-2 cells endogenously express VIP receptor and downstream signaling components including adenylyl cyclase (Hohmann and Tashjian, 1984).

### 2.4 Transfections

All transfections were performed using X-tremeGENE 9 Reagent according to the manufacturer’s protocol with a modification, as described previously (Kim et al, 2018). For all experiments, we first prepared a DNA transfection complex consisting of DMEM, X-tremeGENE 9 Reagent, and plasmid vector. Next, a cell suspension was prepared by trypsinization of cells followed by resuspension in fresh medium, and DNA transfection complex was added directly to the suspension. After mixing, the cell suspension was plated into multiwell plates, and assays were carried out 24 hours later, as described in sections 2.5 and 2.6.

### 2.5 Live cell cAMP measurement

Cytosolic cAMP levels in live cells were monitored using GloSensor cAMP assay, as described previously (Kim et al, 2018; Wang et al, 2022). Briefly, cells were transfected with pGloSensor-22F cAMP plasmid and the cell suspension was seeded on white solid-bottom 96-well plates (Corning or Greiner Bio-One) at a density of 5.0 × 10^4^ cells/well (1.5 × 10^5^ cells/cm^2^). After 24-hour incubation at 37 °C in 5% CO_2_, cells were placed in fresh MEM (without phenol red; supplemented with 2 mM d-luciferin, 20 mM HEPES, and 0.1% BSA; pH = 7.20 ± 0.02; 300 ± 5 mOsmol/L) and were incubated for 2 hours at room temperature. Cells were treated with IBMX (200–500 *μ*M) and were further incubated for 20 minutes at room temperature. Next, cells were stimulated at time 0 with agonists or vehicle, together with nucleotides or vehicle. The emitted luminescence was measured at room temperature with a 1-second integration time at 1.5-minute intervals for a total of 45 minutes.

### 2.6 Live cell β-arrestin-1-PAC1R interaction assay

Agonist-promoted binding of *β*-arrestin-1 to PAC1R was assessed using a luminescent protein complementation assay, as described previously (Misawa et al, 2010; Kim et al, 2018). Briefly, HEK293H cells were transfected with the 2 plasmids (1:1 molar ratio) in suspension and plated on a white clear-bottom 96-well plate (Corning) at a density of 5 × 10^4^ cells/well (1.5 × 10^5^ cells/cm^2^). After 24-hour incubation at 37 °C in 5% CO_2_, cells were placed in fresh MEM (without phenol red; supplemented with 3.2 mM d-luciferin, 20 mM HEPES, and 0.1% BSA; pH = 7.20 ± 0.02; 300 ± 5 mOsmol/L) and incubated for 1 hour at 37 °C. Next, cells were stimulated at time 0 with PACAP or vehicle, together with ATP or vehicle. Luminescence was measured at 37 °C using an LMax^M^II^384^ plate reader at 37 °C with a 2-second integration time at 2-minute intervals for a total of 80 minutes.

### 2.7 Data analyses and statistics

Data shown are means ± SEM. Data obtained in the live cell cAMP measurement and *β*-arrestin-1-PAC1R interaction assays were response versus time curves. Data were analyzed as described previously (Kim et al, 2018; Wang et al, 2022). Briefly, for each time-course curve, we calculated average slopes, beginning at every point and using the next 3 consecutive data points (each average was the mean of the 3 individual slopes). We then selected the maximum slope for each time-course curve, which was then normalized as a fraction of the greatest slope within the experiment. The resultant values were then plotted as a function of agonist concentration.

To assess the time-to-maximum-slope (ie, “lag time”) for each time-course curve, we determined the time elapsed from the addition of agonist (time 0) to the first data point of the maximum slope. This procedure is illustrated in Supplemental Fig. 1.

A 3-parameter sigmoidal equation was fitted to concentration–response data using simultaneous nonlinear regression analysis of multiple data sets, except for Fig. 4, where a 4-parameter variable slope sigmoidal equation was used (GraphPad Prism software). These analyses yielded estimates of minimum signal, EC_50_, and maximum signal. The *F*-statistic (calculated using the extra sum-of-squares *F* test) was used to assess the effect of extracellular nucleotides on EC_50_ and maximum response, by constraining the relevant parameter to be the same between data sets acquired with and without extracellular nucleotide. Differences were accepted as statistically significant at *P* < .05.

## 3 Results

### 3.1 Extracellular ATP enhances adenylyl cyclase activity induced by stimulation of calcitonin receptors

**Fig. 1:**
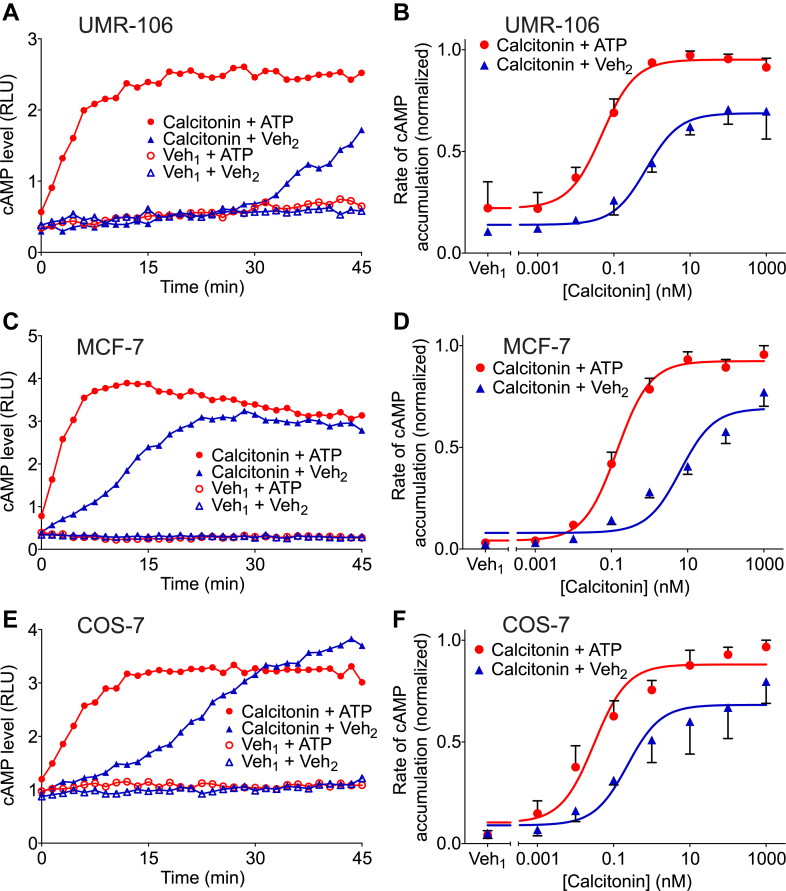
Extracellular ATP enhances calcitonin-induced cyclic AMP (cAMP) accumulation. UMR-106 cells (A, B) and COS-7 cells (E, F) were cotransfected with pGloSensor-22F cAMP biosensor plasmid and a plasmid encoding the calcitonin receptor. MCF-7 cells, which endogenously express calcitonin receptors, were transfected with pGloSensor-22F cAMP biosensor plasmid (C, D). After pretreatment with d-luciferin and 3-isobutyl-1 methylxanthine, cells were stimulated with calcitonin (1 nM) or its vehicle (Veh_1_) in the presence of ATP (1.5 mM) or its vehicle (Veh_2_). (A, C, and E) Illustrate the change in luminescence over time in response to calcitonin (1 nM) or vehicle in the absence or presence of ATP. Each of these time-course graphs shows an individual experiment representative of 3 independent experiments. Luminescence intensity, which corresponds to the level of cytosolic cAMP, was measured from live cells every 1.5 minutes. In (B, D, and F), cells were stimulated with the indicated concentration of calcitonin (or its vehicle, Veh_1_) in the presence of ATP (1.5 mM) or its vehicle (Veh_2_). The maximum rate of cAMP accumulation was determined as the maximum slope of each cAMP level versus time curve. Data were normalized as a fraction of the greatest value of cyclase activity in each experiment. Values are means ± SEM (*n* = 3 independent experiments, each performed in triplicate). The potency of calcitonin in the presence of ATP was increased by an order of magnitude or more as compared with calcitonin alone in MCF-7 and UMR-106 cells. ATP enhanced the maximum response to calcitonin by approximately a third in MCF-7, UMR-106, and COS-7 cells (based on extra sum-of-squares *F* test, Table 1).

We first investigated the effect of extracellular ATP on calcitonin-induced cAMP accumulation. UMR-106 cells were transiently cotransfected with plasmids encoding the calcitonin receptor plus a luciferase-based cAMP biosensor. To suppress cAMP hydrolysis, cells were pretreated with cAMP phosphodiesterase inhibitor IBMX. Cells were then stimulated with calcitonin (1 nM) or its vehicle in the presence of ATP (1.5 mM) or its vehicle. As expected, calcitonin on its own produced a modest time-dependent increase in cAMP levels (Fig. 1A). ATP, although on its own not producing any detectable increase in cAMP levels, markedly enhanced the calcitonin-induced cAMP accumulation.

We also evaluated whether this effect of ATP might vary with calcitonin concentration. Cells were treated with a range of concentrations of calcitonin in the presence or absence of 1.5 mM ATP. Then, the maximal rate of cAMP accumulation under each condition was determined from the time-course data as the greatest slope. In the absence of calcitonin, ATP alone did not induce a significant increase in cAMP accumulation (Fig. 1B, Veh_1_). Calcitonin on its own yielded a sigmoidal concentration dependence curve (Fig. 1B, blue curve) with an EC_50_ of 0.7 nM (Table 1). In the presence of ATP, the potency of calcitonin was increased by about 14-fold. Also, ATP enhanced the maximum response to calcitonin by approximately a third (Fig. 1B, red curve, Table 1, *P* < .001). These data clearly indicate that ATP potentiates calcitonin receptor signaling in cells where the receptor is transiently expressed, and that the increase in signal depends upon the concentration of the orthosteric agonist.

**Table 1:** Summary of parameters for agonist concentration dependence data For each indicated figure panel, 3-parameter sigmoidal equations were fitted simultaneously to the concentration dependence data. Presented are best-fit values ± SEM for pEC_50_ and maximum response. The *F-*statistic (calculated using the extra sum-of-squares *F* test) was used to assess the effect of extracellular ATP or CMP on these values.

Figure Panel	Agent (mM) *or* Receptor	−LogEC_50_	Normalized Maximum
Calcitonin Receptor			

1B	*Vehicle*	9.15 ± 0.21	0.69 ± 0.04
	ATP (1.5)	10.31 ± 0.16∗∗∗	0.95 ± 0.03∗∗∗
1D	*Vehicle*	8.19 ± 0.18	0.68 ± 0.05
	ATP (1.5)	9.86 ± 0.08∗∗∗	0.88 ± 0.03∗∗∗
1F	*Vehicle*	9.64 ± 0.32	0.69 ± 0.04
	ATP (1.5)	10.51 ± 0.18	0.92 ± 0.02∗∗

Calcitonin Receptor-Like Receptor + RAMP1/2/3			

3B	RAMP1	7.98 ± 0.14	0.90 ± 0.04
	RAMP2	6.33 ± 0.14	0.28 ± 0.02
	RAMP3	6.26 ± 0.15	0.47 ± 0.03
3D	*Vehicle*	8.45 ± 0.20	0.66 ± 0.04
	CMP (1.5)	8.97 ± 0.13∗	0.80 ± 0.03∗∗
	ATP (1.5)	8.94 ± 0.10∗	0.94 ± 0.03∗∗∗
3G	*Vehicle*	6.73 ± 0.28	0.57 ± 0.07
	ATP (1.5)	7.12 ± 0.12	0.94 ± 0.04∗∗∗
3J	*Vehicle*	7.09 ± 0.04	0.40 ± 0.04
	ATP (1.5)	7.44 ± 0.03	0.94 ± 0.03∗∗∗

Corticotrophin Releasing Factor Receptor			

5B	*Vehicle*	8.17 ± 0.23	0.63 ± 0.05
	CMP (1.5)	9.89 ± 0.20∗∗∗	0.92 ± 0.05∗∗
	ATP (1.5)	9.73 ± 0.24∗∗	0.86 ± 0.05∗

Pituitary Adenylyl Cyclase-Activating Polypeptide Receptor 1			

6B	*Vehicle*	9.09 ± 0.07	0.92 ± 0.02
	ATP (1.5)	10.00 ± 0.07∗∗∗	0.94 ± 0.02

Vasoactive Intestinal Peptide Receptor 1/2 (VPAC1R/VPAC2R)			

6E	*Vehicle*	9.56 ± 0.05	0.95 ± 0.01
	ATP (1.5)	10.26 ± 0.06∗∗∗	0.92 ± 0.02
6H	*Vehicle*	8.90 ± 0.05	0.94 ± 0.01
	ATP (1.5)	9.42 ± 0.04∗∗∗	0.94 ± 0.01
6K	*Vehicle*	8.35 ± 0.18	0.82 ± 0.05
	ATP (1.5)	9.24 ± 0.09∗∗∗	0.87 ± 0.02

Pituitary Adenylyl Cyclase-Activating Polypeptide Receptor 1 (*β*-Arrestin Binding)			

7B	*Vehicle*	6.86 ± 0.05	1.06 + 0.05
	ATP (1.5)	7.88 ± 0.06∗∗∗	0.90 ± 0.02∗

^∗^*P* < .05, ^∗∗^*P* < .01, ^∗∗∗^*P* < .001 versus corresponding *vehicle* control (*F* test).

To further evaluate the effect of ATP on calcitonin signaling, we investigated whether ATP enhanced calcitonin-induced cAMP accumulation in MCF-7 cells, which endogenously express the calcitonin receptor (Findlay et al, 1981). MCF-7 cells were transiently transfected with a plasmid encoding luciferase-based cAMP biosensor and assayed for ATP effects on calcitonin-stimulated cAMP production as above. Comparable to our findings with UMR-106 cells, ATP by itself showed no effect on intracellular cAMP levels but increased both the observed potency and maximal effect of the orthosteric agonist calcitonin by roughly 50-fold and 30%, respectively (Fig. 1C, D). It follows that the potentiating effect of ATP is not an artifact related to CTR overexpression.

Calcitonin receptor trafficking to the plasma membrane and thus its signaling can be increased by interactions with RAMPs, which are endogenously expressed in many common cell lines (Hay and Pioszak, 2016). To assess whether the ability of ATP to potentiate calcitonin signaling is dependent on the presence of RAMPs, we used COS-7 cells, which are reported to lack endogenous RAMP protein function (Hay and Pioszak, 2016). Because COS-7 cells also lack endogenous calcitonin receptors (Hay et al, 2005), they were cotransfected with plasmids encoding the calcitonin receptor and the cAMP biosensor, and then stimulated with calcitonin (1 nM) or its vehicle in the presence of ATP (1.5 mM) or its vehicle. As expected, calcitonin alone produced a time-dependent increase in cAMP levels. ATP again markedly enhanced the rate of calcitonin-induced cAMP accumulation (Fig. 1E). We also evaluated how this effect of ATP depended on calcitonin concentrations. Again, calcitonin alone yielded a sigmoidal concentration dependence curve (Fig. 1F, blue curve) and ATP shifted the curve to the left, corresponding to a 7-fold increase in potency (Fig. 1F, red curve, Table 1). Consistent with the other 2 cell lines, ATP enhanced the maximum response to calcitonin in COS-7 cells by about a third. Thus, the lack of RAMP activity in these cells does not preclude the effects of ATP. Taken together, the results shown in Fig. 1 demonstrate that extracellular ATP increases agonist potency in cells expressing either endogenous or exogenous calcitonin receptors, and that this increase is not dependent on interaction of the calcitonin receptor with RAMPs.

### 3.2 ATP alters the kinetics of calcitonin-induced cAMP accumulation

Visual inspection of the time course of cellular cAMP accumulation in response to 1 nM calcitonin under control conditions (solid blue triangles in Fig. 1A, C, and E) revealed that the rate of luminescence increase starts relatively slowly and then increases with time. In contrast, a maximal rate is reached sooner when ATP is also present (solid red circles). This ability of ATP to quicken the onset of signaling is not formally factored into the respective concentration–response curves, as values plotted on the Y axes reflect only the maximum slope of luminescence over time under each experimental condition (Fig. 1B, D, and F). Nonetheless these analyses still reveal clear increases in agonist potency and efficacy in the presence of extracellular ATP.

To assess the decrease in signaling latency associated with extracellular ATP, we examined its effect on the lag time between agonist addition and the inflection point at which the maximal rate of signaling commences. As described in section 2 and Supplemental Fig. 1, we evaluated the time required to reach the maximum rate of calcitonin-induced cAMP accumulation in UMR-106, MCF-7, and COS-7 cells using data from the same experiments illustrated in Fig. 1.

**Fig. 2:**
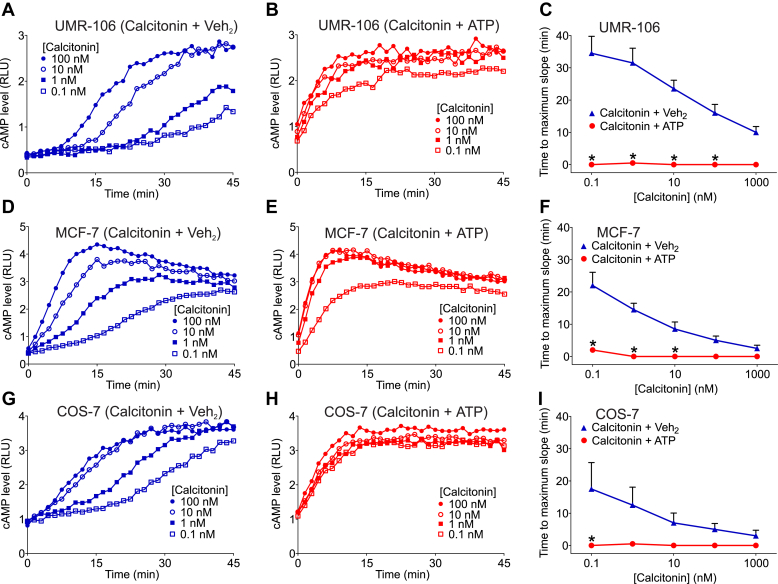
ATP alters kinetics of calcitonin-induced cyclic AMP (cAMP) accumulation. (A, B, D, E, G, and H) UMR-106 (A and B) and COS-7 (G and H) cells were cotransfected with pGloSensor-22F cAMP biosensor plasmid and a plasmid encoding the calcitonin receptor. MCF-7 (D and E) cells were transfected with pGloSensor-22F cAMP biosensor plasmid. Cells were treated with 3-isobutyl-1 methylxanthine. At time 0, cells were stimulated with indicated concentrations of calcitonin in the presence of ATP (B, E, and H; 1.5 mM) or its vehicle (A, D, and G; Veh_2_). Calcitonin alone elevated cAMP levels in a concentration-dependent manner. Notably, this elevation of cAMP levels first exhibited a delay. This lag phase was then followed by a rapid increase in the rate of change in cAMP level. Then, the rate of change in cAMP level peaked (where the maximum slope occurred) before declining, causing cAMP levels to eventually plateau. This delay was more evident at lower concentrations of calcitonin. ATP eliminated this delay, causing the rate of change in cAMP level to peak immediately upon agonist stimulation. (C, F, and I) Delay (quantified as the time-to-maximum-slope) was evaluated for the indicated concentrations of calcitonin in the presence and absence of ATP, as described in Supplemental Fig. 1. Values are means ± SEM (*n* = 3 independent experiments, each performed in triplicate). Note that this figure is derived from the same data as Fig. 1. The asterisk (∗) indicates a statistically significant difference between calcitonin + ATP and calcitonin + Veh_2_ (*P* < .05, based on 2-way analysis of variance and Bonferroni test).

Calcitonin alone at various concentrations produced multiphasic increases in cAMP accumulation (Fig. 2A, D, and G). The rate of cAMP accumulation, which corresponds to a slope in the time-course data, gradually increased following the calcitonin stimulation until a maximum rate was achieved. Notably, the time between calcitonin addition and the attainment of a maximal rate of cAMP accumulation (time to maximum slope) varied with calcitonin concentration; at higher concentrations of the agonist, the time to maximum slope was markedly shorter than that at lower concentrations (eg, Fig. 2A, D, and G; cf. 100 nM vs 0.1 nM). This delay was evident in UMR-106, MCF-7, and COS-7 cells. Interestingly, in the presence of ATP, this delay was essentially eliminated, and the maximum rate of cAMP accumulation was achieved by the first measured time point after stimulation with any concentration of calcitonin (Fig. 2B, E, and H). The diminution in lag time associated with the presence of ATP became less pronounced as agonist concentration was increased, with Bonferroni analysis showing statistically significant differences at calcitonin concentrations from 0.1 to 100 nM in UMR-106 cells, from 0.1 to 10 nM in MCF-7 cells, and at 0.1 nM in COS-7 cells (Fig. 2C, F, and I). These data suggest that ATP accelerates calcitonin binding and/or the transition to maximal activation of adenylyl cyclase in response to receptor occupancy by the agonist.

### 3.3 Potentiation of CGRP and adrenomedullin receptor signaling by ATP

**Fig. 3:**
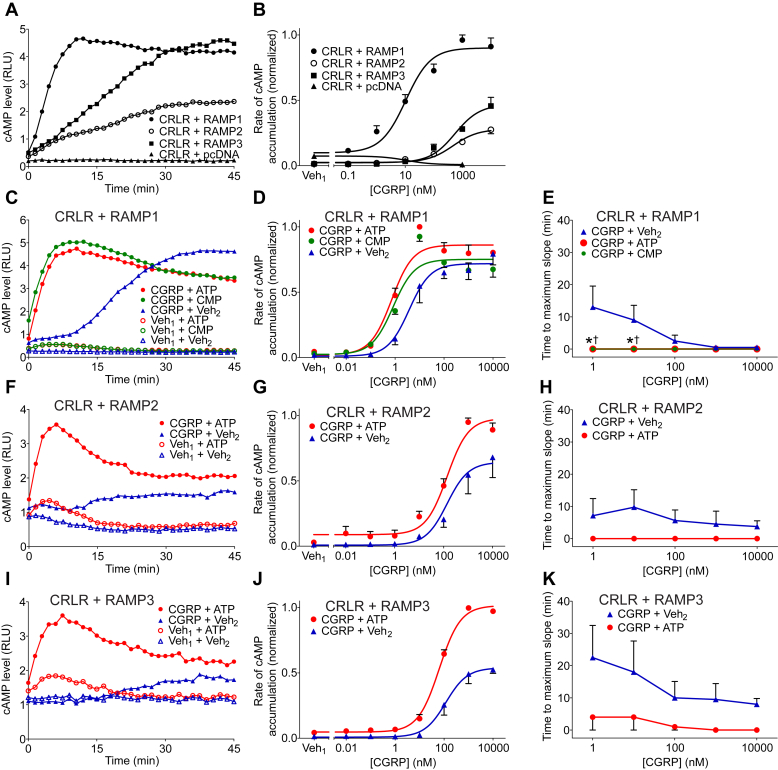
ATP effects on calcitonin gene–related peptide (CGRP) and amylin receptor signaling. (A) COS-7 cells were cotransfected with (1) pGloSensor-22F cyclic AMP (cAMP) biosensor plasmid, (2) a plasmid encoding CRLR, and (3) a plasmid encoding RAMP1, 2, 3, or vector plasmid (pcDNA). At time 0, cells were treated with CGRP (1 *μ*M) or its vehicle (Veh_1_) in the presence of ATP (1.5 mM) or its vehicle (Veh_2_). (A) Illustrates the results of an individual experiment, representative of 3 independent experiments. (B) Cells were stimulated with the indicated concentration of CGRP (or its vehicle, Veh_1_). Adenylyl cyclase activity was determined as the maximum slope of each cAMP level vs time curve. Data were normalized as a fraction of the greatest value of cyclase activity in each experiment. Values are means ± SEM (*n* = 3 independent experiments, each performed in duplicate). (C, F, and I) COS-7 cells were cotransfected with (1) pGloSensor-22F cAMP biosensor plasmid, (2) a plasmid encoding CRLR, and (3) a plasmid encoding RAMP1 (C), RAMP2 (F), or RAMP3 (I). At time 0, cells were stimulated with CGRP (10 nM) or its vehicle (Veh_1_) in the presence of ATP (1.5 mM), CMP (1.5 mM; panel C), or vehicle (Veh_2_). Panels illustrate the results of an individual experiment, each representative of 3 to 4 independent experiments. (D, G, and J) Cells were stimulated with the indicated concentration of CGRP (or its vehicle, Veh_1_) in the presence of ATP (1.5 mM), CMP (1.5 mM; panel C), or vehicle (Veh_2_). Maximum rate of cAMP accumulation was determined as the maximum slope of each cAMP level vs time curve. Data were normalized as a fraction of the greatest value of cyclase activity in each experiment. Values are means ± SEM (*n* = 3-4 independent experiments). CGRP potency in the presence of ATP was increased as compared with CGRP alone for cells coexpressing RAMP1 but not RAMP2 and RAMP3. The maximum response to CGRP was enhanced by ATP in the presence of RAMP1, RAMP2, and RAMP3 (based on extra sum-of-squares *F* test, Table 1). (E, H, K) Time-to-maximum-slope was evaluated for the indicated concentrations of CGRP in the presence of ATP (1.5 mM), CMP (1.5 mM; E), or vehicle (Veh_2_). Values are means ± SEM (*n* = 3-4 independent experiments). The asterisk (∗) indicates significant difference between CGRP + ATP and CGRP + Veh_2_ and the dagger (†) indicates significant difference between CGRP + CMP and CGRP + Veh_2_ (*P* < .05, based on 2-way analysis of variance and Bonferroni test). CRLR, calcitonin receptor-like receptor; RAMP, receptor activity-modifying protein.

RAMP interactions with the calcitonin receptor-like receptor (CRLR) modulate its agonist selectivity by changing its relative binding affinities for calcitonin gene–related peptide (CGRP) and adrenomedullin. In the absence of any RAMP, CRLR exhibits little or no ability to produce cAMP in response to CGRP (McLatchie et al, 1998). To investigate the effects of ATP on CGRP and adrenomedullin receptor signaling, COS-7 cells (which lack endogenous RAMP activity) were cotransfected with (1) a plasmid encoding CRLR, (2) a plasmid encoding RAMP1, 2, 3, or vector plasmid, and (3) a plasmid encoding the luciferase-based cAMP biosensor. First, cells were stimulated with CGRP (1 *μ*M) or treated with an equivalent volume of vehicle (Fig. 3A, B). Confirming the expected lack of RAMP expression in COS-7 cells, CGRP produced little or no increase in cAMP level in CRLR-expressing COS-7 cells in the absence of exogenous RAMP expression. With RAMP1, RAMP2, or RAMP3 coexpression, CGRP induced cAMP accumulation (Fig. 3A). To further evaluate CGRP-induced cAMP accumulation, cells were stimulated with various concentrations of CGRP and the maximum rate of cAMP accumulation under each condition was determined (Fig. 3B). In the presence of RAMP1, CGRP yielded a sigmoidal concentration dependence curve revealing an agonist potency of ∼10 nM (Table 1), consistent with signaling via the CGRP receptor, which is formed in the presence of both CRLR and RAMP1. In the presence of RAMP2 or RAMP3, CGRP potency was 50-fold lower, and maximal cAMP production was less than that in the presence of RAMP1, consistent with the behavior of adrenomedullin 1 and adrenomedullin 2 receptors, respectively (McLatchie et al, 1998).

We next evaluated whether ATP enhanced CGRP-induced cAMP accumulation. COS-7 cells were cotransfected with plasmids encoding (1) CRLR, (2) RAMP1, 2, or 3, and (3) cAMP biosensor. Then, cells were stimulated using CGRP (10 nM) or its vehicle in the presence of ATP (1.5 mM) or vehicle. Again, CGRP at this concentration produced a modest time-dependent increase in cAMP levels. ATP, although having little or no effect on its own, markedly enhanced the CGRP-induced cAMP accumulation regardless of which RAMP isoform was present (Fig. 3C, F, and I). In the presence of ATP, the potency of CGRP was increased 3-fold in cells transfected with CRLR and RAMP1 (Fig. 3D, Table 1). In addition, ATP enhanced the maximum response to CGRP in cells transfected with CRLR together with RAMP1, RAMP2, or RAMP3 by 40%, 65%, and 135%, respectively (Fig. 3D, G, and J, Table 1). Interestingly, ATP had no measurable effect on CGRP potency toward adrenomedullin receptors (ie, CRLR plus RAMP2 or RAMP3), which are poorly activatable by this agonist, whereas effects on maximal signaling were quite pronounced. In contrast to its effects on the behavior of adrenomedullin receptors, ATP measurably increased agonist potency at the eponymous CGRP receptor (ie, CRLR plus RAMP1), whereas its effect on maximal cAMP production was less pronounced.

These data suggest that expression of RAMPs does not either positively or negatively influence the enhancing effect of ATP on CRLR signaling. This, taken together with the observation that ATP enhances calcitonin-induced cAMP accumulation in COS-7 in the absence of RAMP (Fig. 1E, F), implies that RAMPs are not required for the enhancing effect of ATP.

We next assessed whether the pyrimidine nucleotide – cytidine 5-monophosphate (CMP) – could mimic the enhancing activity of ATP on CGRP signaling. As shown in Fig. 3C and D, CMP produced effects like those of ATP in COS7 cells expressing CRLR plus RAMP1.

To assess whether ATP and CMP altered kinetics of CGRP-induced cAMP accumulation, we determined the time to maximum slope using time-course data (see Supplemental Fig. 2). Treatment with CGRP alone resulted in a concentration-dependent delay to the maximum rate of cAMP accumulation, where higher agonist concentrations again were associated with shorter lag times (Fig. 3E, H, and K; blue lines). ATP and CMP eliminated this delay and the maximum rate of cAMP accumulation occurred immediately after stimulation with CGRP (Fig. 3E, H, and K; red and green lines). These data suggest that ATP and CMP potentiate CRLR signaling potency and efficacy, and additionally alter the kinetics of CGRP-induced cAMP accumulation.

**Fig. 4:**
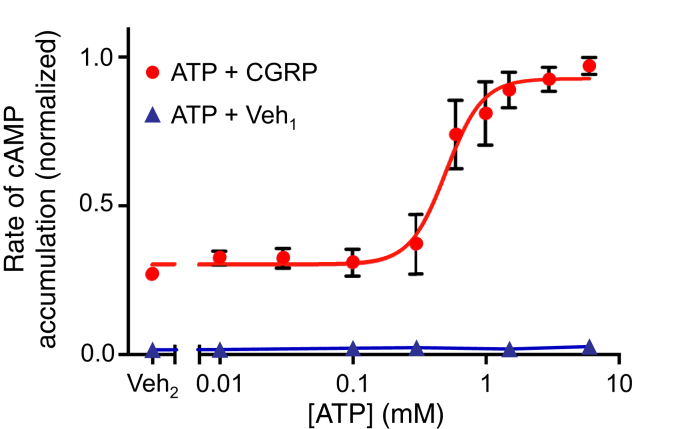
Dependence of potentiation of CGRP signaling on ATP concentration. COS-7 cells were cotransfected with pGloSensor-22F cyclic AMP (cAMP) biosensor plasmid, a plasmid encoding CRLR, and a plasmid encoding RAMP1. At time 0, cells were treated with CGRP (10 nM) or its vehicle (Veh_1_) in the presence of the indicated concentrations of ATP or its vehicle (Veh_2_). Data were normalized as a fraction of the greatest value of cyclase activity in each experiment. Values shown are means ± SEM (*n* = 3 independent experiments, each performed in duplicate). A 4-parameter sigmoidal equation was fitted to the data acquired in the presence of CGRP, yielding an EC_50_ value of 0.5 mM for ATP and a slope factor of 3.2. CGRP, calcitonin gene–related peptide; CRLR, calcitonin receptor-like receptor; RAMP, receptor activity-modifying protein.

To further characterize the potentiation of CGRP receptor signaling, we also examined the effects of a range of ATP concentrations in the absence and presence of 10 nM CGRP, a concentration which reliably showed a potentiating effect (Fig. 4). The EC_50_ of ATP under these conditions was 500 *μ*M. Thus, the concentration of 1.5 mM ATP used in the present study corresponds to a near-maximal potentiating effect. Interestingly, there was notable day-to-day variability in the effects of intermediate ATP concentrations, as indicated by the relatively large error bars associated with points between 100 *μ*M and 1 mM. Also, the data corresponded more closely to a 4-parameter sigmoidal model than a 3-parameter model and yielded a Hill coefficient of 3.2. The mechanisms underlying the observed concentration dependence of ATP are unclear, but both the steepness and variability suggest the possibility of an all-or-none phenomenon.

### 3.4 Extracellular ATP and CMP enhance CRF-induced cAMP accumulation

**Fig. 5:**
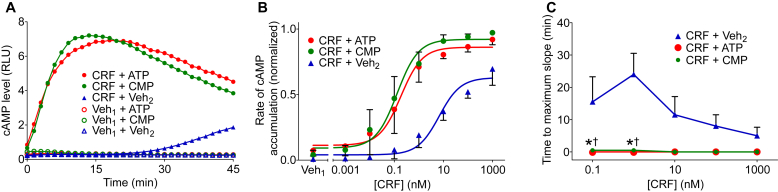
Extracellular ATP and CMP enhance corticotropin-releasing factor receptor (CRF)-induced cyclic AMP (cAMP) accumulation. (A) HEK293H cells were cotransfected with pGloSensor-22F cAMP biosensor plasmid and a plasmid encoding CRF1R. At time 0, cells were stimulated with CRF (1 nM) or its vehicle (Veh_1_) in the presence of ATP (1.5 mM), CMP (1.5 mM) or vehicle (Veh_2_). Panels illustrate the results of an individual experiment, each representative of 3 independent experiments. (B) Cells were stimulated with the indicated concentration of CRF (or its vehicle, Veh_1_) in the presence of ATP (1.5 mM), CMP (1.5 mM) or vehicle (Veh_2_). Maximum rate of cAMP accumulation was determined as the maximum slope of each cAMP level vs time curve. Data were normalized as a fraction of the greatest value of cyclase activity in each experiment. Values are means ± SEM (*n* = 3 independent experiments, each performed in duplicate). pEC_50_ values for CRF in the presence of ATP or CMP were significantly greater than the pEC_50_ for CRF alone. The maximum response to CRF was enhanced by ATP (based on extra sum-of-squares *F* test, Table 1). (C) Time-to-maximum-slope was evaluated for indicated concentrations of CRF in the presence of ATP (1.5 mM), CMP (1.5 mM) or vehicle (Veh_2_). Values are means ± SEM (*n* = 3 independent experiments, each performed in duplicate). The asterisk (∗) indicates significant difference between CRF + ATP and CRF + Veh_2_ and the dagger (†) indicates significant difference between CRF + CMP and CRF + Veh_2_ (*P* < .05, based on 2-way analysis of variance and Bonferroni test).

We next investigated whether ATP and CMP might enhance CRF-induced cAMP accumulation. HEK-293H cells were cotransfected with plasmids encoding CRF1R and the luciferase-based cAMP biosensor. Cells were stimulated with CRF (1 nM) or its vehicle in the presence or absence of ATP (1.5 mM), CMP (1.5 mM), or vehicle. CRF alone produced a time-dependent increase in cAMP levels. ATP and CMP, although producing no detectable increase in cAMP level on their own, markedly enhanced CRF-induced cAMP accumulation (Fig. 5A).

We also examined how this effect of ATP and CMP might vary with CRF concentration. Cells were stimulated with increasing concentrations of CRF in the presence of ATP (1.5 mM), CMP (1.5 mM), or vehicle, and the maximum rate of cAMP accumulation under each condition was determined from the time-course data. CRF alone yielded a sigmoidal concentration dependence curve (Fig. 5B, blue curve). In the presence of ATP or CMP, the measured potency of CRF was increased by ∼30-fold (Fig. 5B, red and green curves; Table 1).

We then assessed whether ATP and CMP altered the kinetics of CRF-induced cAMP accumulation by determining the time to maximum slope using time-course data (Supplemental Fig. 3). With CRF alone, there was a concentration-dependent decrease in the time needed to reach the maximum rate of cAMP accumulation (Fig. 5C; blue line). ATP and CMP essentially eliminated this delay, and the maximum rate of cAMP accumulation was achieved by the first time point taken after CRF stimulation (Fig. 5C, red and green lines). Thus, ATP and CMP potentiate CRF1R signaling and facilitate the onset of CRF-induced cAMP accumulation.

### 3.5 PACAP/VIP receptors are sensitive to ATP but display little hysteretic effect

**Fig. 6:**
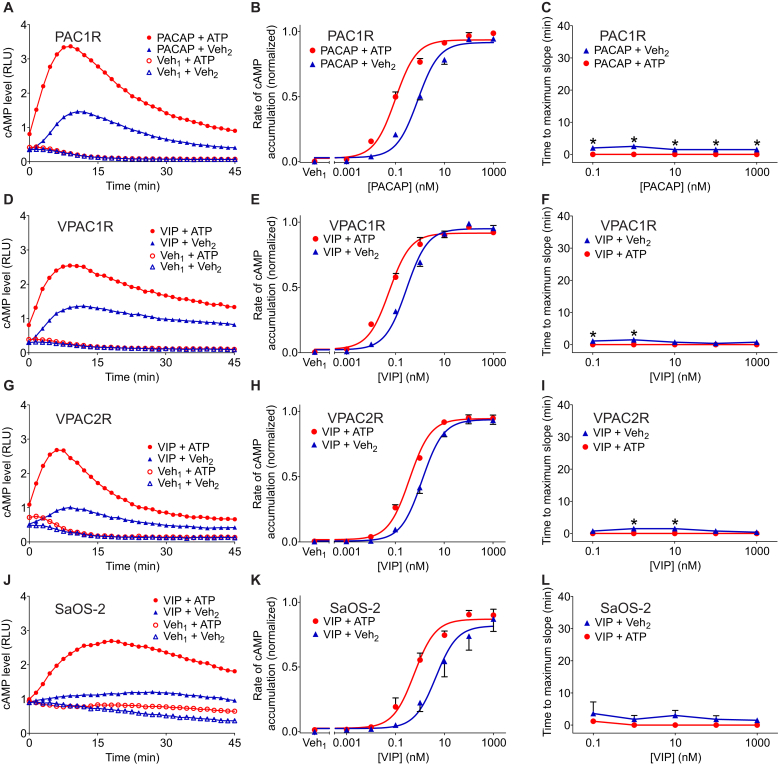
Extracellular ATP enhances cyclic AMP (cAMP) accumulation in response to activation of PAC1R, VPAC1R, and VPAC2R. (A, D, G, and J) COS-7 cells were cotransfected with pGloSensor-22F cAMP biosensor plasmid and a plasmid encoding PAC1R (A), VPAC1R (D), or VPAC2R (G). SaOS-2 cells were transfected with pGloSensor-22F cAMP biosensor plasmid (J). At time 0, cells were stimulated with 0.1 nM PACAP, 0.1 nM VIP, or vehicle (Veh_1_) in the presence of ATP (1.5 mM) or its vehicle (Veh_2_). (B, E, H, and K) Cells were stimulated with the indicated concentration of PACAP, VIP, or vehicle (Veh_1_) in the presence of ATP (1.5 mM) or vehicle (Veh_2_). Maximum rate of cAMP accumulation was determined as the maximum slope of each cAMP level vs time curve. Data were normalized as a fraction of the greatest value of cyclase activity in each experiment. Values are means ± SEM (*n* = 3-5 independent experiments, each performed in triplicate). pEC_50_ values for PACAP or VIP in the presence of ATP were significantly greater than the pEC_50_ values for PACAP or VIP alone (based on extra sum-of-squares *F* test, Table 1). (C, F, I, and L) Time-to-maximum-slope was evaluated for the indicated concentration of PACAP or VIP in the presence of ATP (1.5 mM) or vehicle (Veh_2_). Values are means ± SEM (*n* = 3-5 independent experiments, each performed in triplicate). The asterisk (∗) indicates significant difference between agonist + ATP and agonist + Veh_2_ (*P* < .05, based on 2-way analysis of variance and Bonferroni test). PACAP, pituitary adenylyl cyclase–activating polypeptide; PAC1R, pituitary adenylyl cyclase–activating polypeptide type 1 receptor 1; VIP, vasoactive intestinal peptide; VPAC1R, vasoactive intestinal peptide receptor 1; VPAC2R, vasoactive intestinal peptide receptor 2.

We next focused on the PACAP receptor subfamily, wherein PAC1R is activated by PACAP, and VPAC1R and VPAC2R can be activated by both PACAP and its homolog VIP (Dickson and Finlayson, 2009). COS-7 cells were cotransfected with a plasmid encoding PAC1R, VPAC1R, or VPAC2R and a plasmid encoding the luciferase-based cAMP biosensor. Cells expressing PAC1R were stimulated with PACAP (0.1 nM) or its vehicle in the presence of ATP (1.5 mM) or its vehicle, whereas cells expressing VPAC1R or VPAC2R were stimulated with VIP (0.1 nM) or vehicle in the presence of ATP (1.5 mM) or vehicle (Fig. 6A, D, and G). Both PACAP and VIP produced time-dependent increases in cAMP levels. ATP markedly enhanced PACAP- or VIP-induced cAMP accumulation. We also examined the effects of ATP over a range of concentrations of PACAP or VIP. On its own, each agonist yielded a sigmoidal concentration dependence curve (Fig. 6B, E, and H; blue curves). In the presence of ATP, the concentration dependence curve was shifted to the left, indicating increased agonist potency (Fig. 6B, E, and H; red curves, Table 1).

We then assessed whether ATP altered the kinetics of PACAP- or VIP-induced cAMP accumulation by determining the time to maximum slope using time-course data (Supplemental Fig. 4). Notably, a maximum rate of cAMP accumulation was observed within 3 minutes following the addition of agonist to cells transiently expressing PACAP or VIP receptors, regardless of agonist concentration (Fig. 6C, F, and I; blue lines); this stands in contrast to the behavior seen with other class B GPCRs tested including the calcitonin receptor (Fig. 2), CRLR (Fig. 3), CRF1R (Fig. 5), and PTH1R (Kim et al, 2018), where the maximum rate of cAMP accumulation was typically observed after a delay of 10 minutes or longer at low agonist concentrations. Nevertheless, the simultaneous addition of extracellular ATP together with agonist was associated with a small reduction in this parameter under some of the conditions tested (Fig. 6C, F, and I).

We performed additional experiments to test whether the observed characteristics of PACAP and VIP receptors could be recapitulated in a system where receptors were not overexpressed. SaOS-2 cells endogenously express VPAC1R and downstream signaling components including adenylyl cyclase (Hohmann and Tashjian, 1984; Togari et al, 1997). SaOS-2 cells were transfected with a plasmid encoding the luciferase-based cAMP biosensor and then stimulated with VIP (1 nM) or its vehicle in the presence or absence of ATP (1.5 mM) or its vehicle (Fig. 6J). As anticipated, VIP produced a time-dependent increase in cAMP levels, and ATP enhanced VIP-induced cAMP accumulation. We also examined the effects of ATP over a range of concentrations of VIP. Again, VIP alone yielded a sigmoidal concentration dependence curve, which was shifted to the left in the presence of ATP (Fig. 6K, Table 1). Again, a maximum rate of cAMP accumulation was observed rapidly following the addition of VIP to SaOS-2 cells, regardless of agonist concentration (Fig. 6L). Furthermore, ATP had no appreciable effect on this lag time in SaOS-2 cells.

Overall, despite that PAC1R, VIP1R, and VIP2R do not exhibit the signaling delay evident in other receptors, the presence of ATP increases agonist potency to a degree comparable to its effect at other receptors that were tested.

### 3.6 ATP enhances PACAP-induced β-arrestin-1 recruitment to PAC1R

**Fig. 7:**
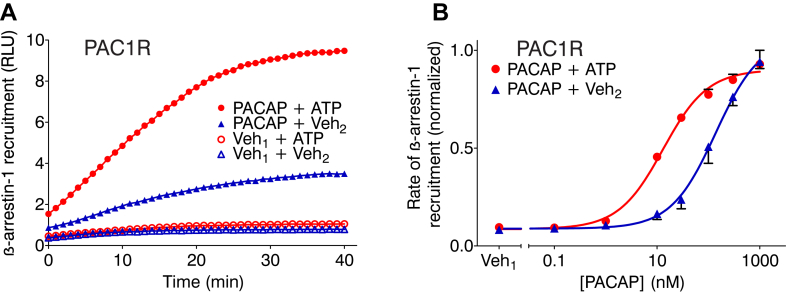
Extracellular ATP enhances PACAP-induced recruitment of *β*-arrestin-1 to PAC1R. (A) HEK293H cells were cotransfected with plasmids encoding N-terminal luciferase fragment-*β*-arrestin-1 and PAC1R-C-terminal luciferase fragment. At time 0, cells were stimulated with the indicated concentration of PACAP (10 nM) or vehicle (Veh_1_) in the presence of ATP (1.5 mM) or its vehicle (Veh_2_). Luminescence intensity, which corresponds to *β*-arrestin-1 recruitment to PAC1R, was then measured from live cells every 2 minutes. Panel illustrates the results of individual experiments, representative of 3 independent experiments each performed in triplicate. (B) Cells were stimulated with indicated concentrations of PACAP or its vehicle (Veh_1_). The maximum rate of *β*-arrestin-1 recruitment was determined from the *β*-arrestin-1 recruitment vs time curves as the greatest slope. Data were normalized as a fraction of the greatest rate of recruitment in each experiment. Values are means ± SEM (*n* = 3 independent experiments, each performed in triplicate). pEC_50_ for PACAP in the presence of ATP was significantly greater than pEC_50_ for PACAP alone (based on extra sum-of-squares *F* test, Table 1). PACAP, pituitary adenylyl cyclase–activating polypeptide; PAC1R, pituitary adenylyl cyclase–activating polypeptide type 1 receptor 1.

To test whether the effects of ATP might extend beyond potentiation of adenylyl cyclase activity, we investigated PACAP-induced recruitment of *β*-arrestin-1 to PAC1R using a split luciferase complementation assay (Misawa et al, 2010). Briefly, HEK293H cells were cotransfected with a plasmid encoding PAC1R fused at its carboxy terminus to a fragment of *Pyrearinus termitilluminans* luciferase, plus another plasmid encoding *β*-arrestin-1 fused at its amino terminus to a complementary luciferase fragment. Cells were then treated with PACAP (10 nM) or its vehicle in the presence of ATP (1.5 mM) or its vehicle. PACAP on its own induced PAC1R-*β*-arrestin-1 coupling (Fig. 7A, solid blue triangles). ATP, although having little effect on its own (open red circles), markedly enhanced PACAP-induced PAC1R-*β*-arrestin-1 coupling (solid red circles).

We further evaluated *β*-arrestin-1 recruitment over a range of PACAP concentrations in the absence and presence of ATP. The maximum rate of recruitment under each set of conditions was determined from the time-course data. PACAP alone stimulated *β*-arrestin-1 coupling in a concentration-dependent manner (Fig. 7B, blue curve). In the presence of ATP, the concentration dependence curve was shifted to the left, indicating an increase in agonist potency (Fig. 7B, red curve; Table 1). Thus, the potentiating effect of ATP on PAC1R signaling extends to the agonist-induced recruitment of *β*-arrestin to the receptor.

## 4 Discussion

### 4.1 Extracellular ATP potentiates class B GPCR signaling

We previously reported on the ability of ATP to alter activity of parathyroid hormone receptor 1 (PTH1R), a class B GPCR (Kim et al, 2018, 2019). In the present study, we demonstrated that extracellular ATP also potentiates signaling mediated by other class B GPCRs including CTR, CRLR, CRF1R, PAC1R, VPAC1R, and VPAC2R. The effect of ATP on these receptors requires the concurrent binding of an orthosteric agonist. Potentiation of agonist signaling could be observed with endogenously expressed receptors (eg, Fig. 1, Fig. 5J, K in the present manuscript, and Kim et al [2018]) and thus is not an artifact of receptor overexpression.

The potentiation of agonist signals by extracellular ATP and CMP is not limited to the adenylyl cyclase pathway, as comparable effects were observed with respect to *β*-arrestin recruitment to PAC1R (Fig. 7), similar to previous findings with PTH1R (Kim et al, 2018). How extracellular ATP alters receptor responsiveness remains unclear; nevertheless, certain possibilities can be ruled out. Importantly, the ability of CMP to mimic the effects of ATP argues against mechanisms based on P2 purinergic receptor activation, ectokinase activity, or hydrolysis of the high-energy *γ*-phosphate bond of ATP.

It is also unclear whether receptors are direct or indirect targets of potentiating molecules. One potential mechanism is direct interaction between ATP and the receptor. This, by extension, would suggest a conserved ATP/CMP interaction site among the class B GPCRs tested. Alternatively, these nucleotides may interact with a common cellular component that in turn enhances signaling by these receptors. It is unlikely that the addition of ATP to the extracellular space would appreciably alter intracellular ATP levels (which are greater than the extracellular concentrations added here; Greiner and Glonek, 2021), and it follows that intracellular class B GPCR binding partners such as G_s_ or *β*-arrestin (Fig. 7) would not be direct targets. Similarly, RAMP proteins, which can regulate class B GPCR trafficking and signaling selectivity, are unlikely to be involved for the reasons outlined above. Conceivably, enhanced adenylyl cyclase sensitivity to activated G_s_ could amplify GPCR signals and thereby engender increased agonist efficacy in terms of cAMP production. Clearly, further studies will be required to reveal the mechanism(s) underlying signal potentiation by ATP.

### 4.2 Potentiating effects vary among different receptors

When we assessed the ability of class B GPCRs to stimulate cAMP production, we found that coaddition of ATP or CMP increased agonist potency (ie, reduced EC_50_) and/or enhanced its maximal effect. As summarized in Table 1, observed increases in agonist potency ranged up to 50-fold, whereas maximal signals as much as doubled. Substantive effects on both parameters were found with CTR and CRF1R. In contrast, potentiation of CGRP effects on CGRP and amylin receptors was characterized predominantly by increases in maximal signal, whereas corresponding effects on the PACAP/VPAC receptor subfamily manifested as increases in agonist potency with no measurable change in maximal signal. The PACAP and VPAC receptors also are distinct from other receptors in terms of their signaling kinetics (see section 4.3). Overall, the variable nature of the potentiating effects of ATP among the receptors tested suggests that these effects are mediated through the receptors themselves.

### 4.3 Kinetic considerations

Any interpretation of the observed potentiation of receptor signaling should consider the temporal complexities in GPCR-stimulated adenylyl cyclase activity uncovered in the present study. Similar to our previous findings with PTH receptor signaling, a period of relatively modest activity was evident prior to the attainment of a maximal rate of cAMP accumulation with several of the receptors examined in the present study (calcitonin receptor, CRLR, and CRF1R). Moreover, this lag time was more pronounced at lower agonist concentrations (eg, Fig. 2C, F, and I). However, PAC1R, VPAC1R, and VPAC2R exhibited little if any latency; the maximum rate of cAMP accumulation was achieved after minimal delay. This variability from one receptor to the next suggests that the time lag is due to a property of the receptor itself. The observed latencies presumably reflect a relatively slow process in the signal transduction pathway that leads to cAMP formation, although what this might be is not yet known. One possible explanation is that the lag period corresponds to some type of major event such as receptor oligomerization (Dong et al, 2010; Harikumar et al, 2012) or internalization (Vilardaga et al, 2011). However, such mechanisms seem unlikely given the rapidity with which maximal rates of cAMP accumulation are reached in the presence of ATP or CMP, ie, the time lag essentially disappears under those conditions. Alternatively, the observed hysteretic effects may stem from a rate-limiting step involving a conformational change in the receptor related to its ability to facilitate activation of G_s_ and/or adenylyl cyclase.

A distinguishing feature of the observed hysteretic effects is that lag time duration decreases as agonist concentration is increased, which suggests an inverse relationship between lag time and agonist binding. The rate of agonist–receptor complex formation would presumably be greater at higher agonist concentrations, as would the rate of re-binding following complex dissociation. Thus, the average occupancy time of each receptor molecule should increase with agonist concentration. It follows that greater time spent by receptors in the apo state would correlate with greater lag times. Conversely, a greater aggregate residence time may lead to more rapid attainment of the “fully activated” state of the signaling system. Regardless of the mechanism underlying the transition from slower to more rapid cAMP production, it appears that exposure to extracellular ATP or CMP facilitates the process in such a way that it is no longer detectable within the timescale of our experiments. Although this loss of hysteresis may contribute to the observed signaling increases with some of the receptors examined in the present study, it cannot account for the observed potentiating effects of ATP on the PACAP subfamily of receptors (ie, PAC1R, VPAC1R, and VPAC2R). Moreover, there is no obvious relationship between the lag time of the response to a given agonist and the magnitude of its EC_50_ shift. Although the underlying mechanism of the enhancing effect of ATP remains to be elucidated, these data are consistent with a process wherein ATP or CMP stabilizes agonist binding to the receptor and/or enhances the ability of the agonist to foster receptor activation.

The present findings reveal a novel mode of signaling modulation by which ATP potentiates signaling at multiple class B GPCRs. For some receptors, there are temporal lags of up to 30 minutes following agonist application before maximal rates of cAMP accumulation are reached. Lag duration decreased with increasing agonist concentration, and ATP virtually abolished this temporal lag. Thus, ATP both increases the potency and/or efficacy of orthosteric agonists at class B GPCRs and reduces latency for adenylyl cyclase activation. As several class B GPCRs are established or emerging therapeutic targets, understanding the mechanisms underlying potentiation should be a priority for future studies.

## Conflict of interest

The authors declare no conflicts of interest.

## References

[bib1] Bodenner D., Redman C., Riggs A. (2007). Teriparatide in the management of osteoporosis. Clin Interv Aging.

[bib2] Born W., Muff R., Fischer J.A. (2002). Functional interaction of G protein-coupled receptors of the adrenomedullin peptide family with accessory receptor-activity-modifying proteins (RAMP). Microsc Res Tech.

[bib3] Burnstock G. (2007). Purine and pyrimidine receptors. Cell Mol Life Sci.

[bib4] Dickson L., Finlayson K. (2009). VPAC and PAC receptors: from ligands to function. Pharmacol Ther.

[bib5] Dong M., Lam P.C., Pinon D.I., Orry A., Sexton P.M., Abagyan R., Miller L.J. (2010). Secretin occupies a single protomer of the homodimeric secretin receptor complex: insights from photoaffinity labeling studies using dual sites of covalent attachment. J Biol Chem.

[bib6] Findlay D.M., Michelangeli V.P., Moseley J.M., Martin T.J. (1981). Calcitonin binding and degradation by two cultured human breast cancer cell lines (MCF 7 and T 47D). Biochem J.

[bib7] Greiner J.V., Glonek T. (2021). Intracellular ATP concentration and implication for cellular evolution. Biology (Basel).

[bib8] Gurevich V.V., Gurevich E.V. (2019). GPCR signaling regulation: the role of GRKs and arrestins. Front Pharmacol.

[bib9] Harikumar K.G., Wootten D., Pinon D.I., Koole C., Ball A.M., Furness S.G., Graham B., Dong M., Christopoulos A., Miller L.J., Sexton P.M. (2012). Glucagon-like peptide-1 receptor dimerization differentially regulates agonist signaling but does not affect small molecule allostery. Proc Natl Acad Sci U S A.

[bib10] Hay D.L., Christopoulos G., Christopoulos A., Poyner D.R., Sexton P.M. (2005). Pharmacological discrimination of calcitonin receptor: receptor activity-modifying protein complexes. Mol Pharmacol.

[bib11] Hay D.L., Pioszak A.A. (2016). Receptor activity-modifying proteins (RAMPs): new insights and roles. Annu Rev Pharmacol Toxicol.

[bib12] Hohmann E.L., Tashjian A.H. (1984). Functional receptors for vasoactive intestinal peptide on human osteosarcoma cells. Endocrinology.

[bib13] Karageorgos V., Venihaki M., Sakellaris S., Pardalos M., Kontakis G., Matsoukas M.T., Gravanis A., Margioris A., Liapakis G. (2018). Current understanding of the structure and function of family B GPCRs to design novel drugs. Hormones (Athens).

[bib14] Kim B.H., Pereverzev A., Zhu S., Tong A.O.M., Dixon S.J., Chidiac P. (2018). Extracellular nucleotides enhance agonist potency at the parathyroid hormone 1 receptor. Cell Signal.

[bib15] Kim B.H., Wang F.I., Pereverzev A., Chidiac P., Dixon S.J. (2019). Toward defining the pharmacophore for positive allosteric modulation of PTH1 receptor signaling by extracellular nucleotides. ACS Pharmacol Transl Sci.

[bib16] Lu V.B., Rievaj J., O'Flaherty E.A., Smith C.A., Pais R., Pattison L.A., Tolhurst G., Leiter A.B., Bulmer D.C., Gribble F.M., Reimann F. (2019). Adenosine triphosphate is co-secreted with glucagon-like peptide-1 to modulate intestinal enterocytes and afferent neurons. Nat Commun.

[bib17] McLatchie L.M., Fraser N.J., Main M.J., Wise A., Brown J., Thompson N., Solari R., Lee M.G., Foord S.M. (1998). RAMPs regulate the transport and ligand specificity of the calcitonin-receptor-like receptor. Nature.

[bib18] Mikolajewicz N., Mohammed A., Morris M., Komarova S.V. (2018). Mechanically stimulated ATP release from mammalian cells: systematic review and meta-analysis. J Cell Sci.

[bib19] Misawa N., Kafi A.K., Hattori M., Miura K., Masuda K., Ozawa T. (2010). Rapid and high-sensitivity cell-based assays of protein-protein interactions using split click beetle luciferase complementation: an approach to the study of G-protein-coupled receptors. Anal Chem.

[bib20] Togari A., Arai M., Mizutani S., Mizutani S., Koshihara Y., Nagatsu T. (1997). Expression of mRNAs for neuropeptide receptors and beta-adrenergic receptors in human osteoblasts and human osteogenic sarcoma cells. Neurosci Lett.

[bib21] Vannabouathong C., Crotty C., Le K., Eurich D., Dyrda P. (2022). Current Utilization Patterns of Glucagon-Like Peptide-1 Receptor Agonists: Report.

[bib22] Vilardaga J.P., Romero G., Friedman P.A., Gardella T.J. (2011). Molecular basis of parathyroid hormone receptor signaling and trafficking: a family B GPCR paradigm. Cell Mol Life Sci.

[bib23] Wang F.I., Ding G., Ng G.S., Dixon S.J., Chidiac P. (2022). Luciferase-based GloSensor cAMP assay: temperature optimization and application to cell-based kinetic studies. Methods.

