# Supplementary material for: Extracellular ATP increases agonist potency and reduces latency at class B G protein-coupled receptors
Source: Mol Pharmacol. 2025 Apr 22;107(6):100040. doi: 10.1016/j.molpha.2025.100040 (PMC12264551; doi:10.1016/j.molpha.2025.100040)
Supplement: Supplementary Figures 1-4 [file mmc1.pdf]

Molecular Pharmacology

MOLPHARM-AR-2024-001049

Supplemental Data

Extracellular ATP increases agonist potency and reduces latency at class B G protein-coupled receptors

Shuying Zhu, Alice Yuan, Tristan Duffy, Brandon Kim, Takeaki Ozawa, S. Jeffrey Dixon, and Peter Chidiac

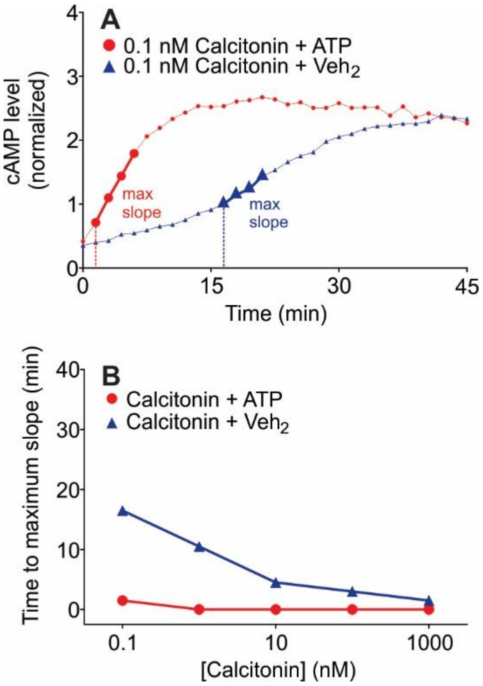

Supplemental Figure 1. Determination of time to maximum slope from cAMP level vs time data. MCF-7 cells were transfected with pGloSensor™-22F cAMP biosensor plasmid. Data illustrate the results of a representative experiment. At time 0, cells were stimulated with calcitonin (0.1 nM) in the presence of ATP (1.5 mM) or its vehicle (Veh$_2$). Luminescence intensity, which corresponds to the level of cytosolic cAMP, was measured from live cells every 1.5 min. Calcitonin elevated the level of cAMP in a time-dependent manner (closed blue triangles). ATP enhanced the rate of cAMP accumulation (closed red circles). Maximum rate of cAMP accumulation was determined from each curve as the maximum slope. Maximum slopes are illustrated in this case with a larger symbol and thicker line. To assess the time-to-maximum-slope (i.e., "lag time"), we determined the time elapsed from the addition of agonist (time 0) to the first data point of the maximum slope. The times to maximum slope in this case are indicated by the dashed lines in panel A. (B) To investigate

the concentration dependence of the time to maximum slope, cells were stimulated with the indicated concentration of calcitonin in the presence of ATP (1.5 mM) or its vehicle (Veh_2).

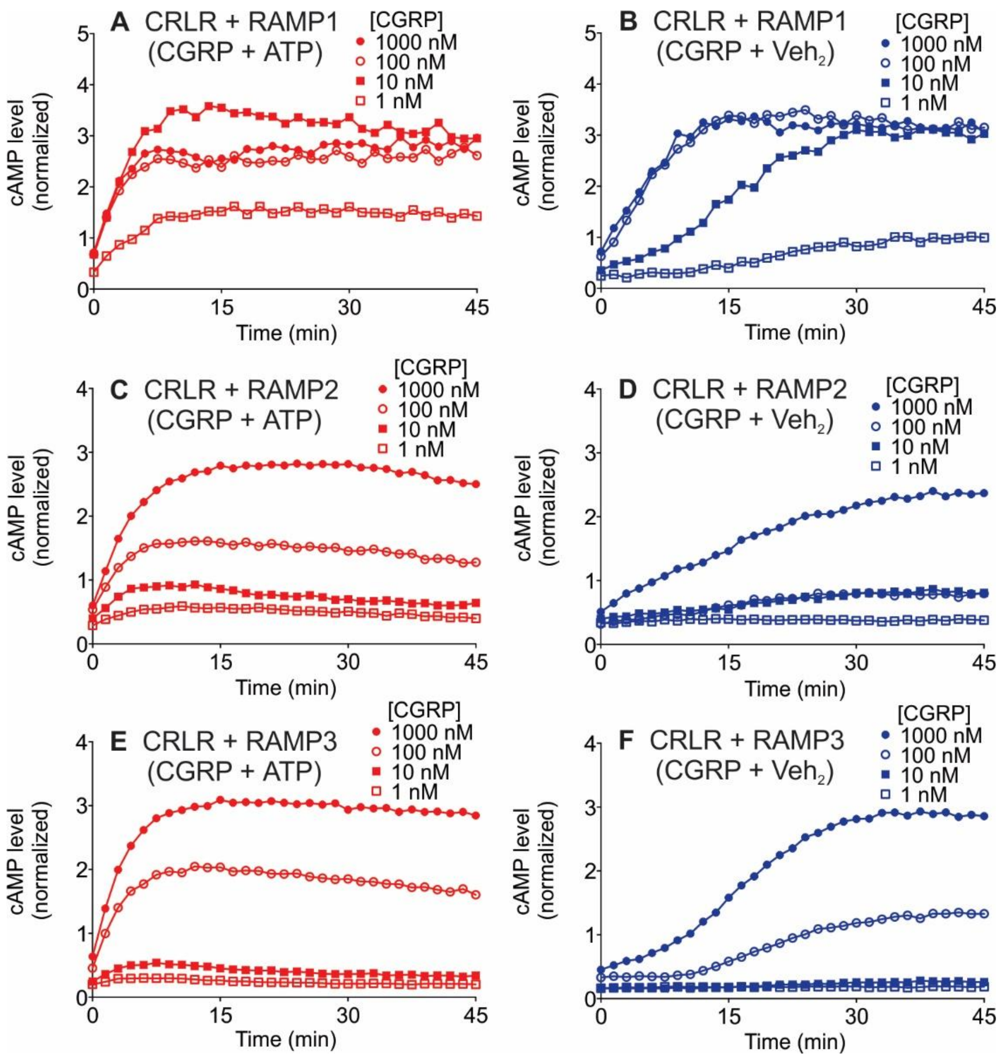

Supplemental Figure 2. Extracellular ATP enhances CGRP-induced cAMP accumulation. COS-7 cells were co-transfected with i) pGloSensor™-22F cAMP biosensor plasmid, ii) a plasmid encoding CRLR, and iii) a plasmid encoding RAMP1 (A and B), RAMP2 (C and D), or RAMP3 (E and F). At time 0, cells were stimulated with indicated concentrations of CGRP in the presence of ATP (A, C and E; 1.5 mM) or its vehicle (B, D and F; Veh_2). CGRP alone elevated cAMP level in a concentration-dependent manner. Notably, this elevation of cAMP level first exhibited a delay. This lag phase was then followed by a rapid increase in the rate of change in cAMP level. Then, the rate of change in

cAMP level peaked (where the maximum slope occurred) before declining, causing cAMP level to eventually plateau. This delay was more evident at lower concentrations of CGRP. ATP eliminated this delay, causing the rate of change in cAMP level to peak immediately upon agonist stimulation. Note that figure is derived from the same data as Fig. 3.

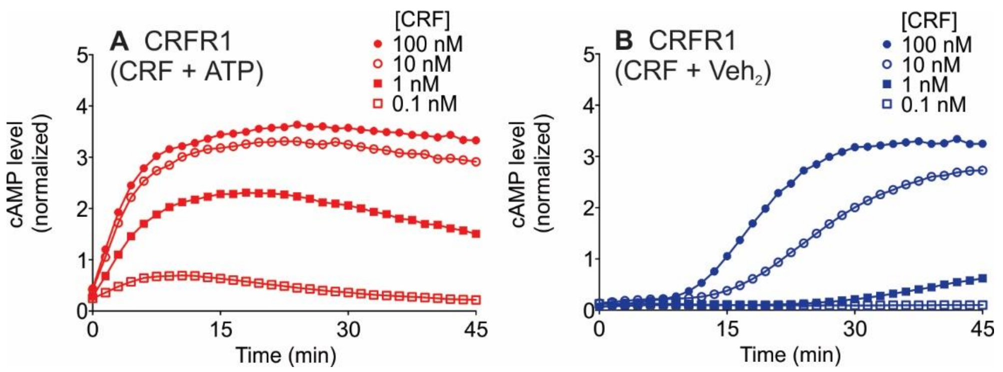

Supplemental Figure 3. Extracellular ATP enhances CRF-induced cAMP accumulation. HEK293H cells were co-transfected with pGloSensor™-22F cAMP biosensor plasmid and a plasmid encoding CRF1R. At time 0, cells were stimulated with indicated concentrations of CRF in the presence of ATP (A; 1.5 mM) or its vehicle (B; Veh_2). CRF alone elevated cAMP level in a concentration-dependent manner. Notably, this elevation of cAMP level first exhibited a delay. This lag phase was then followed by a rapid increase in the rate of change in cAMP level. Then, the rate of change in cAMP level then peaked (where the maximum slope occurred) before declining, causing cAMP level to eventually plateau. This delay was more evident at lower concentrations of CRF. ATP eliminated this delay, causing the rate of cAMP level change to peak immediately upon agonist stimulation. Note that this figure is derived from the same data as Fig. 4.

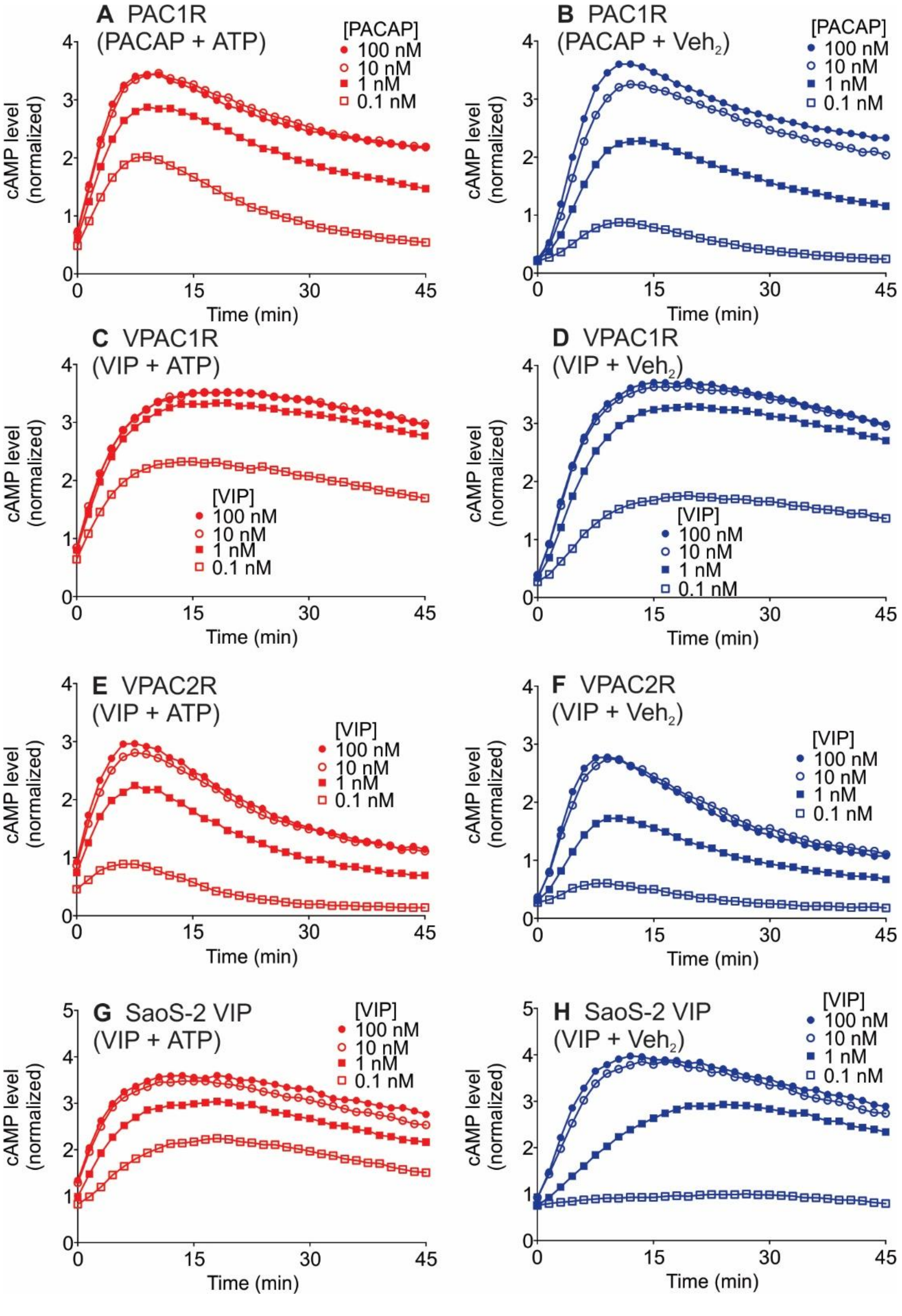

Supplemental Figure 4. Extracellular ATP enhances PACAP- and VIP-induced cAMP accumulation. (A-F) COS-7 cells were co-transfected with pGloSensor™-22F cAMP biosensor plasmid and a plasmid encoding PAC1R (A and B), VPAC1R (C and D), or

VPAC2R (E and F). (G and H) SaOS-2 cells were transfected with pGloSensor™-22F cAMP biosensor plasmid. At time 0, cells were stimulated with the indicated concentration of PACAP, VIP, or vehicle (Veh_1) in the presence of ATP (A, C, E and G; 1.5 mM) or its vehicle (B, D, F and H; Veh_2). PACAP or VIP alone elevated cAMP level in a concentration-dependent manner. Notably, this elevation of cAMP level exhibited a short or no delay; the rate of change in cAMP level peaked (where the maximum slope occurred) shortly or immediately upon agonist stimulation. ATP had little effect on the delay. Note that this figure is derived from the same data as Fig. 5.
